# Detection of *BRCA1*, and *BRCA2* Alterations in Matched Tumor Tissue and Circulating Cell-Free DNA in Patients with Prostate Cancer in a Real-World Setting

**DOI:** 10.3390/biomedicines10123170

**Published:** 2022-12-07

**Authors:** Taylor Ryan McFarland, Vinay Mathew Thomas, Roberto Nussenzveig, Georges Gebrael, Nicolas Sayegh, Nishita Tripathi, Kamal Kant Sahu, Divyam Goel, Benjamin L. Maughan, Deepika Sirohi, Neeraj Agarwal, Umang Swami

**Affiliations:** 1 Division of Oncology, Department of Internal Medicine, Huntsman Cancer Institute, University of Utah, Salt Lake City, UT 84112, USA; 2 Digital Diagnostic Foundation, Cedar City, UT 84720, USA; 3 Division of Pathology, University of Utah, Salt Lake City, UT 84112, USA

**Keywords:** metastatic prostate cancer, BRCA, PARP inhibitor, cancer evolution, cfDNA, next-generation sequencing, genomics

## Abstract

Background: Poly (ADP-ribose) polymerase (PARP) inhibitors are approved for patients with metastatic castration-resistant prostate cancer harboring deleterious or suspected deleterious *BRCA1* and/or *2* mutations. Identifying patients with prostate cancer harboring these mutations may be challenging. Circulating cell-free DNA (cfDNA) provides an avenue for an easier detection of these mutations. Herein, we aimed to evaluate the concordance of *BRCA* mutations in the tumor tissue and cfDNA in patients with metastatic prostate cancer in the real-world setting. Methods: Somatic genomic profiling results were obtained from a clinical cohort of patients at our institution who had at least two samples tested. One of the samples needed to be from either primary or metastatic tissue. Concordance was adjusted to not include mutation types that the cfDNA platforms were not designed to detect. Results: The presence or absence of mutations in the *BRCA* gene was assessed in a total of 589 samples, including 327 cfDNA samples, from 260 patients with metastatic prostate cancer. The median time between the first test and any subsequent test was 22.8 (0.0–232) months. *BRCA* mutation was present in the patient’s original prostate tissue in 23 samples (3.9%) of patients. The adjusted concordance between prostate tumor tissue and cfDNA was 97.9% [95% CI, 95.3–99.1%]. The adjusted concordance between metastatic samples and cfDNA was 93.5% [95% CI, 86.4–97.3%]. Of the patients who had a *BRCA* mutation detected in their prostate tissue, there was a 70% probability of detecting a *BRCA* mutation in the patient’s cfDNA as well. For patients who did not have a detectable *BRCA* mutation in their primary prostate tissue, the probability of detecting a subsequent one later in the disease course was less than 0.9%. Conclusion: There is a high level of concordance between tissue and blood for *BRCA* mutations. Testing cfDNA can provide reliable information on *BRCA* mutational status and is a viable alternative to solid tissue sequencing when unavailable. The development of a new *BRCA* mutation later in the disease course is a rare event.

## 1. Introduction

Deleterious mutations in breast cancer gene 1 (*BRCA1*) and breast cancer gene 2 (*BRCA2*) hamper the homologous recombination pathway, involved in the repair of double-strand breaks in DNA, which can predispose to cancer [1]. Germline mutations in *BRCA1* and *BRCA2* have been associated with an increased incidence of prostate cancer [2,3,4]. Prostate cancers with germline *BRCA1* or *BRCA2* mutations tend to be more aggressive than those without these mutations [5,6,7]. The presence of *BRCA* alterations makes the cancer cell susceptible to poly-ADP (ribose) polymerase (PARP) inhibitors [8,9].

The United States Food and Drug Administration has approved olaparib, a PARP inhibitor for patients with metastatic castration-resistant prostate cancer (mCRPC) harboring deleterious or suspected deleterious germline or somatic homologous recombination repair (HRR) gene mutations who have progressed on abiraterone or enzalutamide [10]. Similarly, rucaparib has been granted accelerated approval for patients with mCRPC harboring deleterious or suspected deleterious germline or somatic *BRCA* mutations who have been previously treated with androgen receptor targeted therapy and taxane-based chemotherapy [10]. These mutations can be detected by genomic profiling of tumor tissue, circulating tumor DNA (ctDNA) or germline testing.

However, genomic profiling to identify patients with mCRPC who are eligible for PARP inhibitors may not be possible in all cases in the real world. For example, in the PROfound study that evaluated olaparib in mCRPC, the results of next-generation sequencing (NGS) could not be obtained from 42% of samples (31% of patients) [11]. The success rate of sequencing decreases as sample age increases, likely due to DNA degradation [11]. Additionally, the sequencing of tumor tissue in patients with mCRPC may be difficult due to the bone predominant nature of the metastatic disease leading to high failure rates of sequencing [12,13]. In such scenario, liquid biopsy can serve as a valuable surrogate for identifying genomic alterations in tumor cells [14,15].

In this study, we use a combination of solid tumor and liquid biopsy sequencing to assess the timing of *BRCA1/2* mutation on a clinical scale in metastatic prostate cancer. We also examine how cfDNA compares to tissue to detect *BRCA* mutations.

## 2. Methods

### 2.1. Patient Selection

This study was performed with approval from the University of Utah Institutional Review Board (IRB #67518, date of approval: 5 September 2013, last approved: 6 December 2022). Patients with prostate cancer from our institution received comprehensive genomic profiling from a Clinical Laboratory Improvement Amendments (CLIA) certified laboratory as part of standard-of-care. The results of these tests were compiled along with clinical information from the electronic health record.

### 2.2. Genomic Profiling

cfDNA testing was performed during routine standard-of-care treatment using a commercially available panel, Guardant360 (Guardant Health, Inc., Redwood City, CA, USA) [16] and FoundationOne Liquid CDx (Foundation Medicine, Inc., Cambridge, MA, USA) [17]. Tumor sequencing was done using FoundationOne CDx (Foundation Medicine, Inc., Cambridge, MA, USA) and Tempus xT (Tempus, Inc., Chicago, IL, USA). All variant types were analyzed, including small mutations, copy number alterations, and gene rearrangements. Variants of unknown significance were excluded.

### 2.3. Adjusted Concordance

There are several limitations of cfDNA testing that complicate concordance assessment. The cfDNA platforms used in this study are limited in their ability to detect gene rearrangements large indels and copy number alterations. Additionally, since germline mutations are usually present throughout the entire body and not just in the tumor, they provide little information about the dynamics and detectability of tumor-specific mutations.

**Figure 1:**
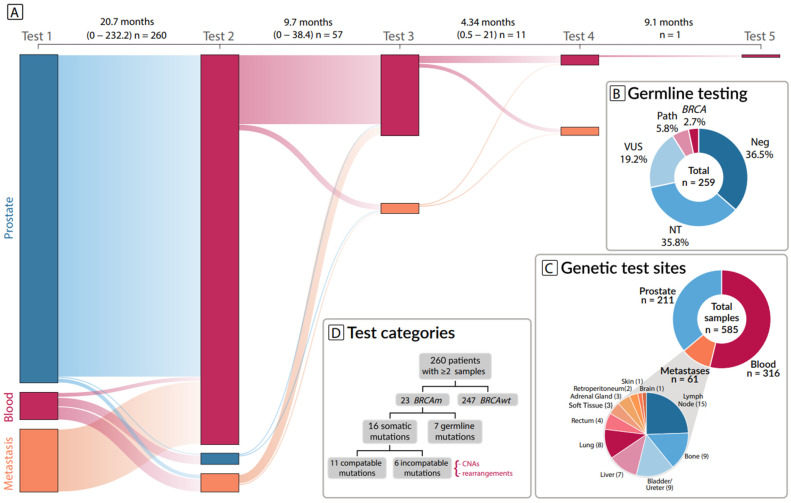
(**A**) Sankey diagram of all samples, the median and range for the time interval between each test is shown at the top. (**B**) Germline testing results, germline *BRCA1/2* mutation (*BRCA*), not tested (NT), variant of uncertain significance (VUS), pathogenic variant (Path), negative result (Neg). (**C**) Sites for the samples with the metastatic samples broken down based on specific location. (**D**) patient selection workflow, copy number alterations (CNAs), *BRCA* mutations (*BRCAm*), *BRCA* wild-type (*BRCAwt*).

For the instances when tissue was being compared to blood, two sets of concordance analyses were performed. The first with no specific samples removed, the second was an “adjusted” group that excluded five patients with the previously mentioned mutations that cfDNA was not capable of detecting. The adjusted group also excluded seven patients with a germline *BRCA* mutation (Figure 1D). Reversion mutations that were detected after treatment with PARP inhibitors were also excluded from all analysis. The calculations of the probabilities of detecting a mutation in cfDNA given a prior result from prostate tissue included germline and non-detectable mutations, but not reversions.

Concordance was calculated using both the *BRCA* mutation positive and negative cohorts. Discordance was when a *BRCA* mutation was not detected in a patient whose tumor was known to have one, or when a patient developed a *BRCA* mutation that was not previously detected.

### 2.4. Software and Analysis

Data analysis was performed using R version 4.0.3 (Boston, MA, USA). Data visualization was done using R and adobe illustrator version 25.2.3 (2021) (San Jose, CA, USA).

## 3. Results

### 3.1. Patient Information

We identified 260 patients with metastatic prostate cancer that had sequencing information from two or more samples for a total of 589 samples. The overall median time between the first test and any subsequent test was 22.8 (0.0–232) months (Figure 1A). The median time between first and any subsequent test for patients with a *BRCA* mutation was 24.25 (0.0–144.9), and for patients without a *BRCA* mutation was 22.8 (0.9–232). The most common type of sample in our cohort was blood (*n* = 327), followed by prostate tissue (*n* = 218), and lastly metastatic tissue (*n* = 61). A detailed breakdown of the metastatic sites is provided in Figure 1C.

Germline *BRCA* status was available for 64.2% percent of patients (Figure 1B). Of the patients with a pathogenic *BRCA* mutation detected in either tissue or blood, all had germline testing information available. *BRCA* mutations were detected in at least one sample in 23 patients (3.9%), seven of which were also found in the patient’s germline.

### 3.2. Concordance Results

**Figure 2:**
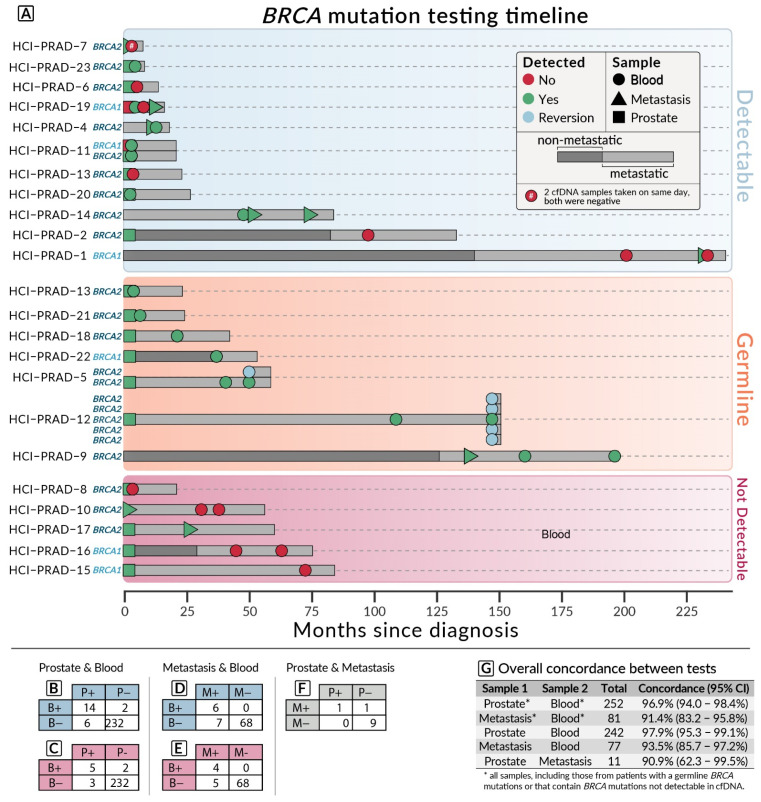
(**A**) Timeline of detection for the 23 patients with *BRCA* mutations. Each grey bar represents one mutation with total length of the bar indicating the time from first diagnosis to last follow up or death, except for the reversion mutations. Note that many of the patients were de novo metastatic. (**B**–**G**) Concordance tables for prostate (P), blood (B), and metastatic (M) samples showing the presence (+) or absence (−) of a *BRCA* mutation. (**B**) Prostate and blood, all samples. (**C**) Prostate and blood, without undetectable mutations. (**D**) Metastasis and blood, all samples. (**E**) Metastasis and blood, without undetectable mutations. (**B**) Prostate and metastasis, all samples.

The adjusted concordance (see methods for details) between prostate tumor tissue and cfDNA was 97.9% [95% confidence interval (CI), 95.3–99.1%] with only two instances of a *BRCA* mutation detected in cfDNA but not prostate tissue (Figure 2C,G). The concordance between metastatic samples and blood was 93.5% [95% CI, 86.4–97.3%] (Figure 2D,G). The results of all concordance analyses are provided in Figure 2B–G.

The probability that a *BRCA* mutation will be detected on the cfDNA testing given that their prostate sample contained a *BRCA* mutation was 70%. The probability that a *BRCA* mutation would be found in a patient’s cfDNA given that no *BRCA* mutation was found in their prostate tissue (i.e., they developed a *BRCA* mutation that was not detected in their primary sample) was less than 0.9%.

Reversion mutations that were determined to restore *BRCA2* function were found in two patients. Both were found in cfDNA taken after they had progressed on treatment with a PARP inhibitor.

Two other patients developed a mutation that was detected in cfDNA but not in their original prostate tumor. One of these patients already had an existing *BRCA2* mutation along with a heavy mutational burden from a germline MutS homolog 2 (*MSH2)* mutation in prostate biopsy but additional *BRCA1* was detected in the cfDNA (HCI-PRAD-11). The other, though not detected in the primary sample, was a *BRCA1* mutation found 2.43 months later in patient HCI-PRAD-19. This same *BRCA1* mutation was also detected in a lung metastasis sample taken 9.87 months later after it was found in cfDNA. Of the patients with only metastatic tissue available, none had a *BRCA* mutation that was found exclusively in cfDNA (Figure 2E,G).

## 4. Discussion

Our study indicates high concordance of genomic analysis in tumor tissue and cfDNA. There was a high probability that cfDNA testing would detect a *BRCA* mutation if present in the primary prostate or metastatic site tissue. Conversely, the chance of finding a *BRCA* mutation in the cfDNA sample when the mutation was not present in the tumor tissue was low. These data also suggest that the development of *BRCA* mutations is an early event in the ontogeny of prostate cancer.

These data are consistent with the prior studies [14,18,19]. Tukachinsky and colleagues reported that with tumor tissue as reference, the positive percentage agreement for *BRCA1/2* on cfDNA was 93% [14]. Schweizer and colleagues evaluated the concordance of the presence of DNA damage repair (DDR) gene mutations in archival primary tissue compared to cell-free circulating tumor DNA and metastatic tissue [18]. In the 51 patients included for final analysis, concordance in DDR gene mutation status was noted to be 84% [18]. In the PROfound study, 81% of cfDNA samples yielded an NGS result, and with tumor tissue as a reference, the positive and negative percentage agreement for *BRCA* and ataxia-telangiectasia mutated (*ATM)* mutations was 81% and 92%, respectively [19].

Although cfDNA represents a viable alternative for somatic testing, successful cfDNA analysis depends on a high cfDNA fraction. There exist few potential challenges such as clonal hematopoiesis of indeterminate potential, inability to differentiate between somatic and germline mutations, pre-analytic, analytic, and post-analytic limitations which are seldom reported by commercial platforms.

In our study, there was a 70% probability of detecting a *BRCA* mutation in cfDNA if present in the tumor tissue. However, we did not adjust this analysis for clinical factors such as high PSA levels or the volume of disease, which could have possibly increased the test sensitivity. The apparent explanation for the *BRCA* mutations present in cfDNA but absent in the primary prostate tissue could be due to the emergence of subclones due to treatment selection [14,18]. Though not included in the concordance analysis, reversion mutations were seen in two of our patients. These mutations switch tumor cells from HRR deficient to HRR proficient through somatic base substitutions or insertions/deletions [20]. Reversion mutations promote resistance to PARP inhibitors by restoring HRR function in tumor cells and enabling them to repair DNA damage caused by PARP inhibitors [21]. Similarly, Quigley and colleagues reported a restored function of *BRCA2* function in two patients who progressed on olaparib and talazoparib [22].

Another possible reason for detecting *BRCA* mutations in cfDNA alone compared to primary prostate tissue could be differences in the genomic composition across tumor foci in the prostate [18]. Gundem and colleagues demonstrated that lethal prostate cancer arises from low-grade tumors whereby specific subclones within the primary tumor develop metastatic potential [23]. There would likely be discordance in the mutational profile if the prostate tumor clone selected for sequencing was different than the one that metastasized [18]. This hypothesis would explain the finding in one of our two patients with a *BRCA* mutation in cfDNA not seen in the primary prostate tissue. Subsequent genomic analysis from lung metastasis in this patient demonstrated the same *BRCA* mutation in cfDNA. Interestingly, none of the patients with only metastatic tissue available for genomic analysis had a *BRCA* mutation found exclusively in cfDNA.

Given the high concordance of the prostate and metastatic tissue with cfDNA, we conclude that if tissue is unavailable or inadequate for sequencing, profiling of cfDNA is a valuable alternative for detecting *BRCA* mutations.

Our results had limitations encountered with real-world studies, such as its retrospective nature, single-institutional experience, a relatively small number of patients with a *BRCA* mutation and matched primary and cfDNA testing, and lack of external validation. Additionally, the genetic heterogeneity between prostate tumor foci was not considered when assessing mutational concordance. In summary, our results indicate a low probability of detecting a *BRCA* mutation in the cfDNA if it is not present in the primary tissue. A high concordance was observed for *BRCA* mutational status between tumor tissue and cfDNA.

## Data Availability

The data generated in this study are not publicly available as they could compromise patient privacy but are available upon reasonable request from the corresponding author.
